# Stress causing psychosomatic illness among nurses

**DOI:** 10.4103/0019-5278.50721

**Published:** 2009-04

**Authors:** Pratibha P. Kane

**Affiliations:** Maharashtra Medical Research Society, Pune, India

**Keywords:** Burnout, nursing shortage, nursing stress, psychosomatic illness, shift work

## Abstract

Stress in nurses is an endemic problem. It contributes to health problems in nurses and decreases their efficiency. Documenting the causes and extent of stress in any healthcare unit is essential for successful interventions

**Aim:**

Establishing the existence and extent of work stress in nurses in a hospital setting, identifying the major sources of stress, and finding the incidence of psychosomatic illness related to stress.

**Materials and Methods:**

This study used a questionnaire relating to stressors and a list of psychosomatic ailments. One hundred and six nurses responded and they were all included in the study. Stressors were based on four main factors: work related, work interactions, job satisfaction, and home stress. The factors relating to stress were given weights according to the severity. The total score of 50 was divided into mild, moderate, severe, and burnout.

**Results:**

Most important causes of stress were jobs not finishing in time because of shortage of staff, conflict with patient relatives, overtime, and insufficient pay. Psychosomatic disorders like acidity, back pain, stiffness in neck and shoulders, forgetfulness, anger, and worry significantly increased in nurses having higher stress scores. Increase in age or seniority did not significantly decrease stress.

**Conclusion:**

Moderate levels of stress are seen in a majority of the nurses. Incidence of psychosomatic illness increases with the level of stress. Healthcare organizations need to urgently take preemptive steps to counter this problem.

## INTRODUCTION

Stress affecting nurses across the globe has been convincingly documented in the literature for more than 40 years.[1] Nurses' environment include an enclosed atmosphere, time pressures, excessive noise or undue quiet, sudden swings of from intense to mundane tasks, no second chance, unpleasant sights and sounds, and long standing hours.[2]

Nurses are trained to deal with these factors but chronic stress takes a toll when there are additional stress factors like home stress, conflict at work, inadequate staffing, poor teamwork, inadequate training, and poor supervision. Stress is known to cause emotional exhaustion in nurses and lead to negative feelings toward those in their care.[3]

It is important to identify the extent and sources of stress in a healthcare organization to find stress management strategies to help the individual and the environment. Stress in nurses affects their health and increases absenteeism, attrition rate, injury claims, infection rates, and errors in treating patients.[4]

Unless the healthcare setups acknowledge the problem and take preemptive steps to tackle the growing menace of chronic stress, personnel costs will keep rising and add to the already soaring costs of care. Nurses' absenteeism, turnover, and sickness significantly increase the cost of employment in healthcare units.

## MATERIALS AND METHODS

There are very few studies in India on stress in nurses. In1981, Grey-Toft developed an instrument called the “Nurses Stress Scale.”[3] This scale described 34 situations that could cause stress for nurses. The present study has modified and translated the questionnaire to Marathi to suit the nurses in these hospitals so that their major causes of stress could be identified. Questions related to sexual harassment, addiction, and substance abuse were omitted as most of the nurses in this study come from lower middle class conservative homes. Both job stress and home-related stress factors were included in the study.

A pilot study was carried out by giving these questionnaires to the nurses-in-charge and four senior nurses.

This questionnaire was given to all 120 nurses who worked in two hospitals managed by a private foundation. One hundred and six of them replied and the data were collected.

Total number of stress factors was 27. They were categorized under: work related (8), interaction at work place (7), job satisfaction (5), and home-related stress (7). Each stress factor was graded as 1, 2, or 3 according to its potential to cause stress. Thus, the total factor score was 27 and the total weighted score was 50. Stress factors and severity of stress were graded [Table 1].

**Table 1:** Severity of stress

Grading	Factor score	Weighted score
Mild stress	1–6	Less than 13
Moderate stress	6–13	13–25
Severe stress	13–20	26–37
Burnout	More than 20	More than 37

Analysis was carried out in two ways:

1. Average (mean) number of factors and average weight – present by the illness or symptoms.
  In case of means, unpaired *t*-test is used to compare the means of the two groups of illness/symptom present or absent. *P*-value is given. If *p* < 0.05, the difference between the means is significant.
2. Cross tables – categories of stressors and weight by presence/absence of illness or symptoms. In this case; percentage of illness/symptom in a particular category of stressor or weight is given. From the increase or decrease in the percentage, one can infer the significance. χ^2^ test is applied to test whether the difference is real or by chance [Tables 2 and 3].

**Table 2:** Modified table of statistically significant parameters derived from t-tests (Stress Scale 27 factors)

Parameter		Average number of factors/stressors	*P*-value
Acidity	Present	9.0000	0.020
	Absent	7.5208	
Backache	Present	9.0400	0.035
	Absent	7.6964	
Stiffness of the shoulders	Present	9.0870	0.037
	Absent	7.7500	
Memory problem	Present	10.8750	0.001
	Absent	7.8778	
Getting angry	Present	9.4524	0.004
	Absent	7.5938	
Worrying	Present	9.2407	0.003
	Absent	7.3846	

**Table 3:** Modified table of statistically significant parameters derived from t-tests (stressors with weight 50 factors)

Parameter		Average stress weight	*P*-value
Acidity	Present	17.1379	0.031
	Absent	14.5417	
Backache	Present	17.4000	0.023
	Absent	14.6786	
Memory problem	Present	20.6250	0.001
	Absent	15.1333	
Getting angry	Present	18.4286	0.001
	Absent	14.3438	
Worrying	Present	17.8148	0.001
	Absent	14.0385	
Hb < 10	Yes	19.3571	0.025
	No	15.3889	

## RESULTS

Stress levels were studied in 106 nurses from all units of the hospitals.

Fifty-six percent of the staff has more than 10 years of experience. Age and experience wise, this is a senior workforce [Tables 4 and 5]. Workplace stress should decrease with age and experience and development of skills but this fact was not statistically supported.

**Table 4:** Demographic variables

Age	No.	%	Marital status	No.	%
Less than 25	24	22.6	Married	75	70.8
25–34	42	39.7	Single	23	21.7
35+	40	37.7	Separated/divorced	3	2.8
Sex			Widowed	5	4.7
Women	102	94.8			
Men	4	2.9			
*n* = 106					

**Table 5:** Demographic variables

Qualifications	No.	%
B.Sc.	3	2.7
ANM/GNM	22	20.8
Private course	81	76.5
Work experience		
Up to 4years	18	16.9
5–9 years	28	26.4
10–19 years	51	48.2
20+	9	8.5
Employment status		
Permanent	96	90.6
Contract	4	3.8
Temporary	6	5.6
Type of nursing unit		
Wards	54	50.9
Intensive care/emergency	39	36.8
Operating room	13	12.3
*n* = 106		

73.59% of the nurses suffer from significant stress varying in severity [Table 6].

**Table 6:** Severity of stress in nurses

Stress score	No.	%
Mild (1–13)	28	26.42
Moderate (14–25)	70	66.04
Severe (26–37)	8	7.55
Burnout (> 37)	0	-
*n* = 106		

Major causes of stress that were studied are listed in Tables 7 and 8.

**Table 7:** Work stressors affecting nurses

Work related	No.	%
Not finishing work in time	83	78.3
Backache due to standing for long hours	65	61.3
Shortage of staff	62	58.5
Overtime	31	29.2
Dealing with cardiac arrest/emergencies	29	27.4
Problematic patients/death	24	22.6
Handling patient relatives	23	21.7
Night duty	22	20.8
**Interactions**		
Troublesome relatives	72	67.9
Troublesome patients	60	56.6
Fear of getting infected	30	28.3
No cooperation between nurses	6	5.7
No subordinate support	5	4.7
Doctors do not attend to emergencies in time	5	4.7
Male nurses treated badly	2	1.9
**Job satisfaction**		
Want extra training other than the present	66	62.3
Dissatisfied with salary	64	60.4
Need for training	60	56.6
Insecurity of the job	15	14.2
No job satisfaction	9	8.5

**Table 8:** Home stressors affecting nurses

Home stress		
Dependant relatives	56	52.8
Work disturbs home life	39	36.8
Home stresses more than work	21	19.8
Sole earning member	20	18.9
Need of crèche	15	14.2
No family support	10	9.4
Husband with a drinking problem	9	8.4

The nursing profession is in the middle of the most crippling nursing shortages in its history. By 2020, the workforce will be 20% below requirements. In this study, excessive workload was a major cause of stress and emotional exhaustion.

Back pain due to standing for long hours, lack of exercise, and shifting patients can also decrease efficiency and increase absenteeism. Stiffness in the neck and shoulders seen in the nurses is largely due to continuous tensing of muscles due to stress.

Sixty-six percent of the nurses were interested in training for new skills and 60% desired more training for their present job. Ongoing training and job rotation are yet not an established initiative taken up by HR mangers in hospitals.

Sixty percent of the nurses are not satisfied with their existing salary and benefits.

Fear of exposure to acquired immunodeficiency syndrome (AIDS) and Hepatitis while treating infected patients is a cause of stress, especially in younger nurses who are not trained to protect themselves, taking universal precautions for all patients.

Most of them have a permanent employment status. Therefore, the stress of job insecurity does not affect them.

Home stress contributes significantly to the stress faced by nurses [Table 8]. Their home life is disturbed due to night shifts, overtime, transport delays, and difficulty in getting leave. Worry about children and their studies not being properly supervised are common. Nurses look after the home, cooking, cleaning, etc as they cannot afford domestic help.

The psychosomatic illnesses that were statistically significant in this study are highlighted in Tables 9 and 10.

**Table 9:** Nurses suffering ill health

Physical symptoms	No.	%
Headache	64	60.5
Acidity	58	54.5
Backache	50	47.2
Stiffness in neck and shoulders	46	43.4
Stomach ache	12	11.3
Fainting	9	8.5
Constipation	8	7.5
Blood pressure	4	3.8
Dysmenorrhea	3	2.8
Other gynecological problems	2	1.9
Diabetes	1	0.9
Loose motions	1	0.9
Asthma	1	0.9
Hamoglobin 10 gm% or less	34	32.1
*n* = 106		

**Table 10:** Nurses suffering ill health

Emotional symptoms	No.	%
Tiredness	43	40.6
Crying	23	21.7
Forgetfulness	16	15.1
Anger	42	39.6
Worry	54	50.9
Depression	15	14.2
Loneliness	21	19.8
*n* = 106		

Psychosomatic illness is a disorder that affects the body and the mind. These illnesses have emotional origins causing physical symptoms. Chronic stress is responsible for 90% of these illnesses.

In spite of 60% of the nurses complaining of headache, it was not statistically proved to correlate with increasing level of stress. It could be due to lack of sleep because of the dual responsibility of work and home.

Acidity affects 62% of the nurses. Anemia is seen in 32% of the staff. This may be because of erratic meal times, missing meals because of overwork, and faulty eating and excessive consumption of tea and coffee during the night shift.

From this, study we can infer that acidity, anemia, backache, and stiffness in the neck and shoulders are related to stress at home and workplace. Emotional symptoms of forgetfulness, getting excessively angry, and worrying also significantly affect the nurses in this study [Tables 2 and 3].

## DISCUSSION

Stress is experienced when demands made on us outweigh our resources. A moderate level of stress or “Eustress” is an important motivating factor and is considered normal and necessary. If stress is intense, continuous, and repeated, it becomes a negative phenomenon or “Distress,” which can lead to physical illness and psychological disorders.[6] Psychosomatic illnesses are disorders that involve both the body and the mind. These illnesses are mental or emotional in origin and have physical symptoms. Running hospitals as businesses has changed the working pattern of nursing as a whole. Stress levels are on the rise and little is being done about assessing this malady and actively managing its effects. Nurses are expected to give sensitive quality patient care, have patience, and help disposition and, at the same time, save costs and increase efficiency by keeping a rapid throughput.

Nurses are the backbone of any healthcare unit. The pressures of overtime and long working hours create a work–personal life imbalance, which begins to affect the health of the employees. Other factors such as long commuting hours and chaotic traffic conditions adding to their stress affect the employee's efficiency and effectiveness. It can undermine the employee's relationship at home as well as on the job. This can have a negative influence on their physical and emotional health and lead to psychosomatic disorders. Economic loss to the organization due to errors, wrong decisions, wrong choice, lack of attention, and injury are some of the serious effects of chronic stress.[7] The trend of nurses working overtime started with downsizing of organizations and the trend to have only skeletal manning. Absenteeism is compensated by others doing overtime. This increases the take-home pay but is likely to injure their health. Such nurses experience severe stress and require more sick leaves. This risk increases with the length of overtime.

All nurses have to do shift work or attend emergencies at night. The stress of shift work can also aggravate health conditions and lead to heart disease or digestive disorders.[8] Fatigue can lead to error, injury, and carelessness. Long hours are a source of depression, low morale, and low motivation. Shift workers are on the job in the evening or on weekends and they sleep during the day. Hence, they often miss out on social or family activities.

There is a stark difference in causes of stress in nurses in developed countries and in India. Nurses in India are poorly remunerated compared to the world standards. For the kind of intense work that the nurses do, the salary and benefits are not adequate. Rewards not proportional to workload is a source of great stress as it is difficult to have decent standards of living based only on their basic salary.

Lack of professional respect and recognition by authorities and doctors is the major cause of dissatisfaction in nurses abroad. Poor relationship with physicians was related to musculoskeletal disorders, which is seen as the most important reason for nurses leaving hospitals. Lack of autonomy, poor participation in patient care due to lack of sufficient knowledge, and empowerment deprives them from job satisfaction.

Nurses in India are mainly from the lower economic strata and have low educational qualifications. Their main motivators are salary and benefits to support their home and maintain a decent standard of living. Shortage of staff makes them easily succumb to increasing their pay package by doing excessive hours of overtime at the expense of their health. They have limited access to claims and compensation for occupational hazards.

Many studies of stress in nurse in developed countries have shown chronic stress as a major contributor to suicide or suicidal thoughts, smoking, excessive coffee consumption, and alcohol intake.[9]

Resurfacing of repetitive problems and feeling of the work never getting done added to stress in this study. Turnover for skilled nurses is instigated by internal, on the job factors, which cause dissatisfaction and stress (poor salary, lack of recognition, workplace bullying) and a desire to leave. Replacement is mostly with less skilled staff, which increases the responsibility and load of the remaining skilled staff. Shortage of staff increases the burden of non-nursing jobs, like shifting patients, picking up food trays, making beds, and even filing and keeping record.

Poor nutrition leading to anemia contributes to the poor health of the nurses in this study. Meals are usually not taken in time. Missing breaks to finish work also increased stress. Shift work can interfere with regular eating and digestive circadian rhythm. This could lead to acidity and other stomach problems. However, digestive problems also could be caused by the tendency for excessive consumption of tea or coffee in the night shift.

In the absence of doctors, nurses are on the front line and have to face verbal abuse from patients and relatives for issues that may not be directly connected to their work. Physical violence and aggressiveness is also on the rise in patients and their relations. Demanding patients and their relatives can cause conflict and lead to more stress. Patients' expectations from nurses are sometimes unreasonable and they tend to be aggressive. No training is given to them to deal with confrontation.

Stress-related illness is not imaginary. It is very tricky to diagnose and treat. The key is to look for a source of stress that the person is not coping with.

Chronic stress decreases motivation. It can lead to increased absenteeism and increased turnover and attrition rates. Thus, it is mandatory for healthcare organizations to address this issue urgently. There is urgent need for proactive stress management, especially preventive strategies, as are encouraged in the industry and IT sector. There is need for coping techniques like team building, counseling, learning assertiveness, and communication skills, which should be taught to all nurses, even incorporated in their training curriculum.[2]

Elimination of all stressors is a utopian goal. Effective solutions can be found, like increasing skills, enriching work, and increasing the participation of nurses in the organization. Adequate staffing, which reduces job stress and overtime, could lead to improved efficiency along with cost effectiveness.[10]

### An ounce of prevention is worth a pound of cure:[11]

Every organization should assess the magnitude of stress and analyze it to recognize the need for action. This is also called a “stress audit.” Earlier, stress was viewed as a personal problem to be tackled at an individual level with palliative or remedial measures. Now, the approach is to be proactive, with emphasis on prevention and elimination rather than treatment. Improving the quality of work life of nurses may go a long way to decrease attrition.

This study was inspired by the work of Professor Tom Cox, Amanda Griffith, and Professor Sue Cox, who are committed to the vision of a healthy and productive workforce. I hope this study has made a small contribution to the achievement of that vision.

## CONCLUSION

Seyle's research was the first to demonstrate a correlation between stress and illness.[12] Stress is a slow and insidious malady, which is affecting the healthcare industry, and there is dearth of research on this important topic in developing countries. Psychosomatic illness in nurses needs to be researched further to make policy decisions that will improve the work-life balance for nurses.

Recognition, participation, and continuous training go a long way in retaining skilled staff and preventing a “skill hole.” Organizations must be sensitive to the dual stress of home and work faced by nurses.

Healthy organizations are associated with open management styles and employee empowerment. Organizational climate and values are seen as important, with provision of social support, feedback, and shared rewards as potential strategies for stress reduction.[13]

All hospitals should feel responsible for the well-being of their workforce as it proportionately improves safety standards of their patients. Nurses' positive attitude to their work markedly increases patient satisfaction and patient loyalty. Downsizing as a remedy to cutting costs can adversely affect healthcare delivery and contribute indirectly to the soaring costs of treatment.
